# Supplementary material for: Maillard Reaction Intermediates and Related Phytochemicals in Black Garlic Determined by EPR and HPLC Analyses
Source: Molecules. 2020 Oct 7;25(19):4578. doi: 10.3390/molecules25194578 (PMC7583006; doi:10.3390/molecules25194578)
Supplement: Supplementary file 1 [file molecules-25-04578-s001.pdf]

Sample Garlic

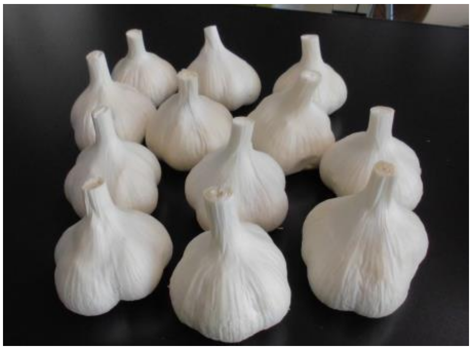

S-1. Garlic bulbs

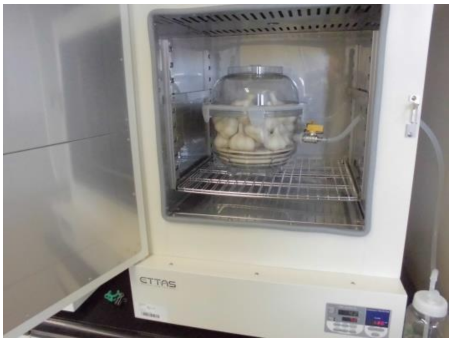

S-1a. Garlic bulbs in an incubator

S-2
Supplemental data

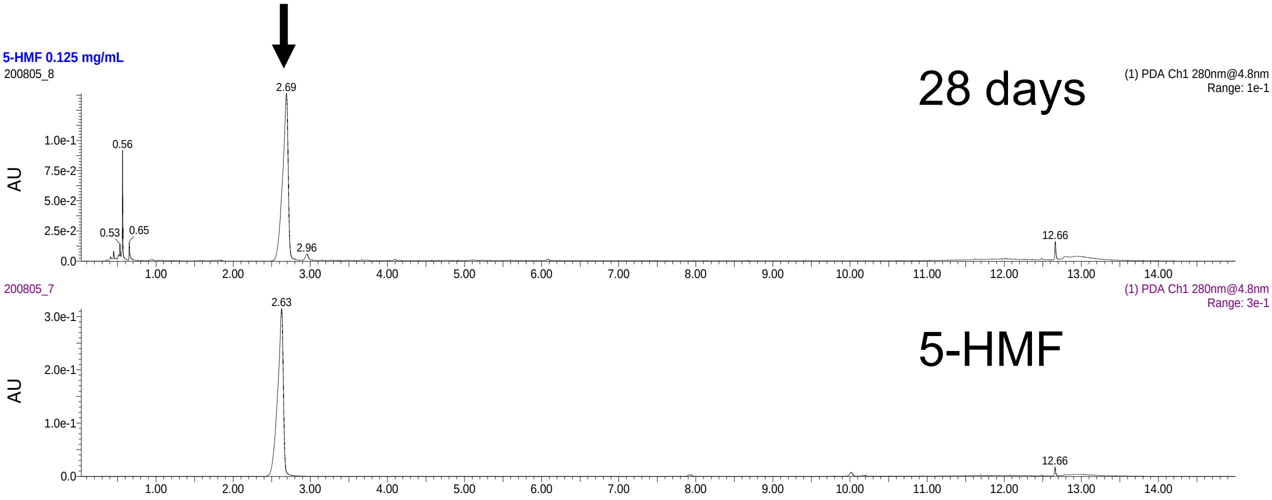

UHPLC-Tof-MS analysis
280 nm chromatogram

S-3
Supplemental data

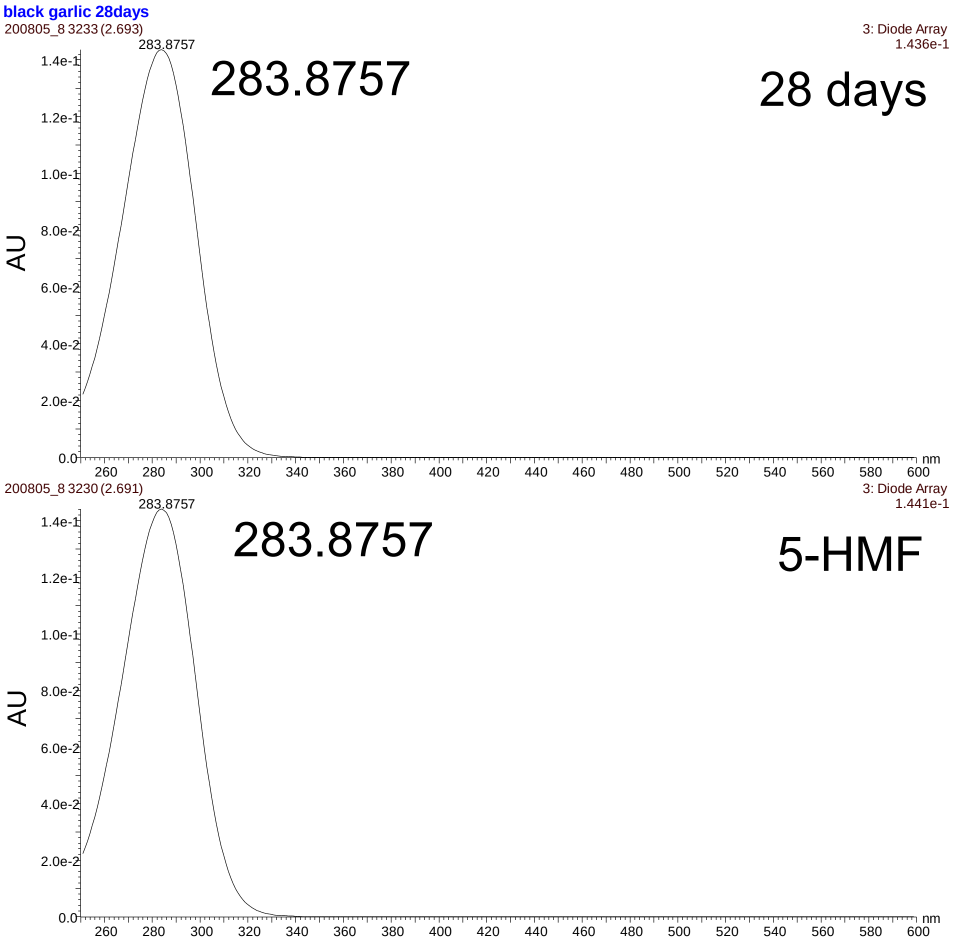

UHPLC-Tof-MS analysis
Max absorption at the peak

S-4
Supplemental data

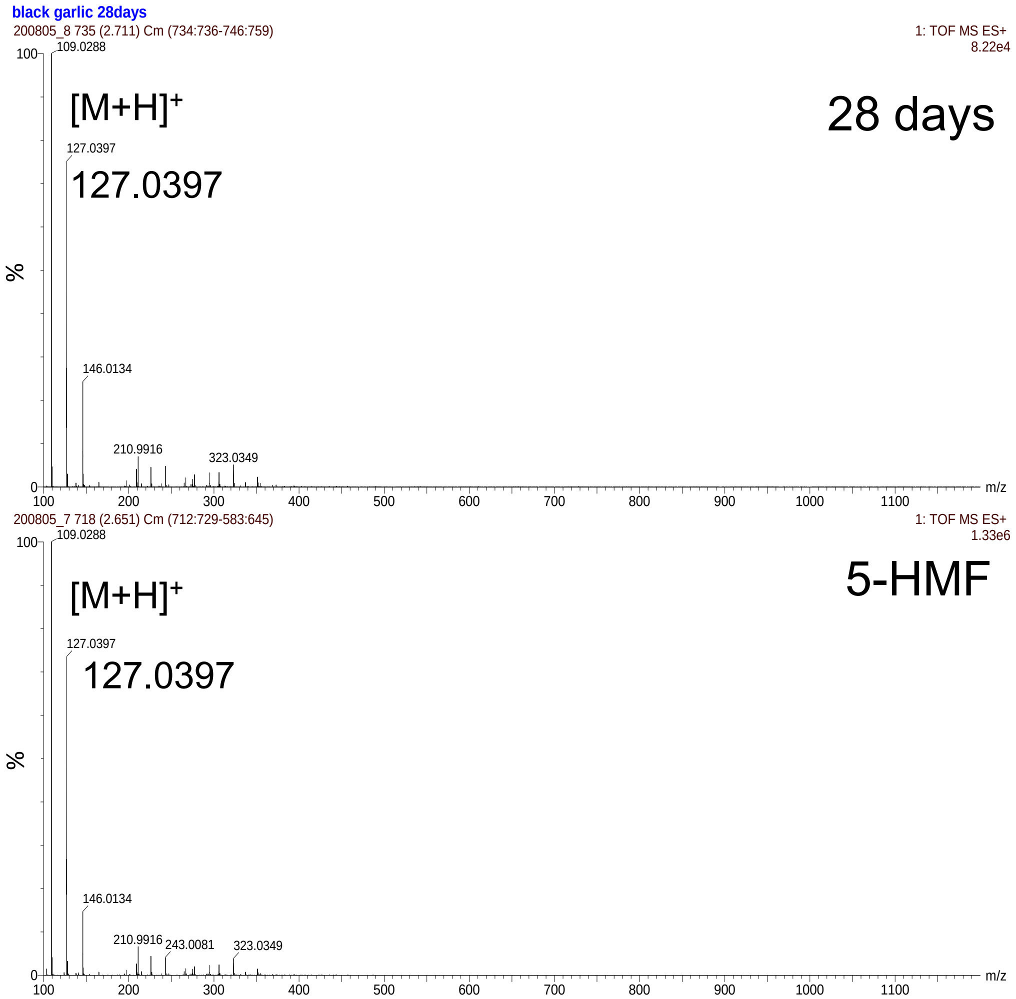

UHPLC-Tof-MS analysis
MS chromatogram
